# Effects of Anthocyanin and Flavanol Compounds on Lipid Metabolism and Adipose Tissue Associated Systemic Inflammation in Diet-Induced Obesity

**DOI:** 10.1155/2016/2042107

**Published:** 2016-06-06

**Authors:** Roel A. van der Heijden, Martine C. Morrison, Fareeba Sheedfar, Petra Mulder, Marijke Schreurs, Pascal P. H. Hommelberg, Marten H. Hofker, Casper Schalkwijk, Robert Kleemann, Uwe J. F. Tietge, Debby P. Y. Koonen, Peter Heeringa

**Affiliations:** ^1^Department of Pathology and Medical Biology, University Medical Center Groningen, University of Groningen, 9713 GZ Groningen, Netherlands; ^2^Top Institute Food and Nutrition, 6709 AN Wageningen, Netherlands; ^3^Department of Metabolic Health Research, Netherlands Organisation for Applied Scientific Research (TNO), 2333 CK Leiden, Netherlands; ^4^Department of Epidemiology, University Medical Center Groningen, University of Groningen, 9713 GZ Groningen, Netherlands; ^5^Department of Pediatrics, University Medical Center Groningen, University of Groningen, 9713 GZ Groningen, Netherlands; ^6^Avans University of Applied Sciences, 5223 DE Breda, Netherlands; ^7^Molecular Genetics, University Medical Center Groningen, University of Groningen, 9713 GZ Groningen, Netherlands; ^8^Department of Internal Medicine, Maastricht University Medical Centre, Maastricht University, 6229 HX Maastricht, Netherlands

## Abstract

*Background.* Naturally occurring substances from the flavanol and anthocyanin family of polyphenols have been proposed to exert beneficial effects in the course of obesity. We hypothesized that their effects on attenuating obesity-induced dyslipidemia as well as the associated inflammatory sequelae especially have health-promoting potential.* Methods.* Male C57BL/6J mice (*n* = 52) received a control low-fat diet (LFD; 10 kcal% fat) for 6 weeks followed by 24 weeks of either LFD (*n* = 13) or high-fat diet (HFD; 45 kcal% fat; *n* = 13) or HFD supplemented with 0.1% w/w of the flavanol compound epicatechin (HFD+E; *n* = 13) or an anthocyanin-rich bilberry extract (HFD+B; *n* = 13). Energy substrate utilization was determined by indirect calorimetry in a subset of mice following the dietary switch and at the end of the experiment. Blood samples were collected at baseline and at 3 days and 4, 12, and 20 weeks after dietary switch and analyzed for systemic lipids and proinflammatory cytokines. Adipose tissue (AT) histopathology and inflammatory gene expression as well as hepatic lipid content were analyzed after sacrifice.* Results.* The switch from a LFD to a HFD lowered the respiratory exchange ratio and increased plasma cholesterol and hepatic lipid content. These changes were not attenuated by HFD+E or HFD+B. Furthermore, the polyphenol compounds could not prevent HFD-induced systemic rise of TNF-*α* levels. Interestingly, a significant reduction in* Tnf* gene expression in HFD+B mice was observed in the AT. Furthermore, HFD+B, but not HFD+E, significantly prevented the early upregulation of circulating neutrophil chemoattractant mKC. However, no differences in AT histopathology were observed between the HFD types.* Conclusion.* Supplementation of HFD with an anthocyanin-rich bilberry extract but not with the flavanol epicatechin may exert beneficial effects on the systemic early inflammatory response associated with diet-induced obesity. These systemic effects were transient and not observed after prolongation of HFD-feeding (24 weeks). On the tissue level, long-term treatment with bilberry attenuated TNF-*α* expression in adipose tissue.

## 1. Introduction

The high incidence of obesity is accompanied by a dramatic increase in metabolic and cardiovascular disorders [1, 2]. Compelling evidence links these disorders to chronic metabolic inflammation [3, 4]. The adipose tissue (AT) and in particular its dysfunction associated with obesity are an important contributing factor to this type of systemic inflammation [5, 6].

The primary role of the AT is to store energy in the form of lipids (triglycerides) for later usage during nutrient deprivation. By storing excess lipids the adipocyte expands, thereby releasing anorexic signals to inhibit energy intake [7]. However, when overconsumption persists, the maximum lipid storage capacity of adipocytes is reached and lipids can no longer be cleared from the circulation, resulting in elevated systemic lipid levels (hyperlipidemia) [8]. In parallel, excessive adiposity also triggers the production of proinflammatory cytokines by adipocytes [9], leading to recruitment of leukocytes to the AT [10].

In a normal inflammatory response, inflammation resolves once the inflammatory trigger has been eliminated. However, in the case of obesity-associated inflammation, the inflammatory triggers in the AT persist, and enhanced recruitment of leukocytes to the AT is observed [11]. With increasing numbers of infiltrating leukocytes, locally produced cytokines may be released into the circulation, thereby contributing to a state of chronic systemic low-grade inflammation (LGI), frequently observed in obese patients [12]. LGI may then promote endothelial activation and leukocyte adhesion in different vascular beds of the body, thereby driving the development of various vascular disorders in the periphery like sclerosis in the aorta or kidney.

Thus, as inflammation in the AT plays a pivotal role in the onset of many obesity-associated metabolic and vascular diseases, attenuating obesity-induced disturbed lipid metabolism and/or the consequent AT inflammation may alleviate or even prevent occurrence of obesity-related pathologies.

In the context of disease prevention, the potential beneficial effects of naturally occurring substances like polyphenols on lipid metabolism and/or consequent inflammation have gained particular interest. Polyphenol compounds belonging to the anthocyanin and flavanol subclasses [13] especially are thought to exert beneficial effects. Anthocyanins have been shown to have various health-promoting lipid-modulating and anti-inflammatory effects [14]. For example, these compounds have been shown to lower plasma cholesterol levels [15], exhibit anti-inflammatory properties [16, 17], or improve adipocyte function [18]. Similarly, flavanol species, especially the catechins, have been shown to exert anti-inflammatory and antioxidative effects* in vivo *[19, 20].

We hypothesized that supplementation of high-fat diets with epicatechin or anthocyanins may have a dual effect in the prevention of metabolic disorders: it may attenuate dyslipidemia and/or obesity-associated inflammation. To address this hypothesis, C57BL/6J mice were fed a high-fat diet (HFD) or a HFD supplemented with epicatechin or a bilberry extract, at established doses [21, 22], for 24 weeks and monitored for lipid metabolism and parameters of local (adipose tissue) and systemic inflammation.

## 2. Materials and Methods

### 2.1. Animal Experiments

C57BL/6J male mice (Charles River, JAX Laboratories, France) were purchased at the age of 6 weeks and randomly divided over four groups. Mice were individually housed in a temperature-controlled room under a 12 h light-dark cycle with* ad libitum *access to water and food. All groups received a control LFD (10 kcal% fat) (Research Diets Inc., NJ, USA) for 6 weeks. Hereafter (*t* = 0) three groups of mice were switched to either a HFD (45 kcal% fat) or HFD supplemented with 0.1% w/w of epicatechin (ChromaDex Inc., Irvine, USA) referred to as the HFD+E group or 0.1% w/w of the standardized bilberry (*Vaccinium myrtillus* L.) extract Mirtoselect which contains 36% of anthocyanins (Mirtoselect, Indena SAS, Paris, France) referred to as the HFD+B group. Data on diet-induced obesity (DIO) were compared to age-matched LFD controls. All HFD types were manufactured at Research Diets Inc. A schematic representation of the experimental design is provided as Supplementary Figure  1 in Supplementary Material available online at http://dx.doi.org/10.1155/2016/2042107. Food intake was monitored between 7-8 and 15-16 and 23-24 weeks, averaged for daily intake (g), and corrected for caloric intake (Supplementary Figure 2).

All experimental procedures were performed with approval of the University of Groningen Ethics Committee for Animal Experiments (DEC 6141B), in accordance with the Dutch law on animal experimentation.

Reference data on the LFD and HFD phenotype, namely, body weight, hepatic lipid, AT histopathology, and inflammation (Figure 5), were published previously but are included here as well to illustrate the effects of DIO and as control for the HFD+E and HFD+B groups [23].

### 2.2. Blood Collection

Blood samples were obtained from mice after 6 h fasting (8 am–2 pm) by puncturing the saphenous vein of the left hind leg. Samples were collected in EDTA-coated capillary tubes (Microvette 300 KC, Sarstedt BV, Etten-Leur, Netherlands) and directly put on ice. Samples were spun down at 3000 rpm, and plasma was aspirated and stored at −80°C. Serial blood samples were collected 2 weeks prior to (baseline) and 3 days and 4, 12, and 20 weeks after dietary switch from the same mice.

### 2.3. Serial Plasma Lipid and Cytokine Analyses

Plasma cholesterol, triglycerides, and free fatty acid (FFA) levels were determined by commercially available kits, according to the manufacturer's instructions (cholesterol: Cholesterol CHOD-PAP, Roche, Woerden, Netherlands; triglycerides: Hitachi, Roche, Woerden, Netherlands; FFA: Diagnostic Systems, Holzheim, Germany).

Plasma TNF-*α*, mouse KC (mKC; CXCL1), IFN-*γ*, and IL-6 levels were measured using a Meso Scale Discovery (Gaithersburg, USA) 10-Plex MULTI-SPOT Mouse Cytokine Assay for plasma, according to the manufacturer's instructions. Plasma concentrations of IL-1*β*, IL-5, and IL-12p70 were under the detection limit of the assay. Plasma serum amyloid A (SAA) was measured by ELISA (Tridelta, Maynooth, Ireland) according to the manufacturer's instructions.

### 2.4. Metabolic Analyses* In Vivo*

Subgroups of mice (*n* = 6–8/group) were placed individually in indirect calorimetric cages (LabMaster TSE Systems, Bad Homburg, Germany) from 3 days prior to till 3 days after the dietary switch (6 days; short-term effect) and after 23 weeks (4 days; long-term effect). Following an initial 24 h acclimatization period, mice were monitored every 13 minutes for the duration of the measurement. The respiratory exchange ratio (RER = VO_2_/CO_2_) was used to estimate the relative contribution of fat and carbohydrates to whole-body energy metabolism in mice. In addition, physical activity and food intake were closely monitored for the duration of the measurements.

### 2.5. Sacrifice Procedure and Organ Processing

After 24 weeks mice were anesthetized (2% isoflurane in O_2_) and whole blood samples were collected by cardiac puncture for EDTA plasma isolation. Three visceral adipose depots (gonadal, mesenteric, and perirenal) were isolated, weighed, partly snap-frozen in liquid nitrogen, and partly fixed in 10% formalin. Frozen samples were stored at −80°C, and formalin fixed samples were processed as described previously and embedded in paraffin [24]. Livers were dissected as a whole, weighed, and then partially stored in paraffin and partially snap-frozen in liquid nitrogen. Approximately 300 mg of the liver was snap-frozen for assessing liver lipid content.

### 2.6. Adipose Tissue Histopathological Assessment

Four *μ*m thin paraffin sections were stained by hematoxylin and eosin according to procedures described previously [24]. Quantification of the number of crown-like structures (CLS) was performed as described previously [23].

### 2.7. Adipose Tissue Gene Expression Analyses

Total RNA was isolated from approximately 300 mg of adipose tissue from the gonadal AT depot using RNeasy Lipid Tissue Mini Kit (Qiagen, Westburg, Leusden, Netherlands) according to the manufacturer's instructions. Procedures for subsequent cDNA synthesis, primer sequences, and RT-qPCR analyses can be found elsewhere [23].

### 2.8. Liver Lipids and TG-Profiling

Approximately 300 mg of snap-frozen liver was homogenized, and total intrahepatic lipids were extracted according to the Bligh and Dyer method [25]. Intrahepatic free cholesterol (FC) and total cholesterol (TC) were quantified using commercially available kits (FC: DiaSys Diagnostic Systems GmbH, Holzheim, Germany; TC: Roche). Besides hepatic TG quantification (Hitachi, Roche, Netherlands), liver homogenates were also analyzed by gas chromatography after transmethylation to determine specific hepatic fatty acid composition. Specific procedures are described elsewhere [26].

### 2.9. Statistical Analyses

Differences between the experimental groups at one time point were analyzed by a nonparametric ANOVA with Dunn's* post hoc* test comparing all groups with HFD. Analyses were performed in GraphPad (version 5.00 for Windows, GraphPad Software, San Diego, CA, USA). Data are expressed as means ± SEM. The level of significance was set at *p* < 0.05.

## 3. Results

### 3.1. Polyphenols Do Not Alter Diet-Induced Obesity/Adiposity

**Figure 1:**
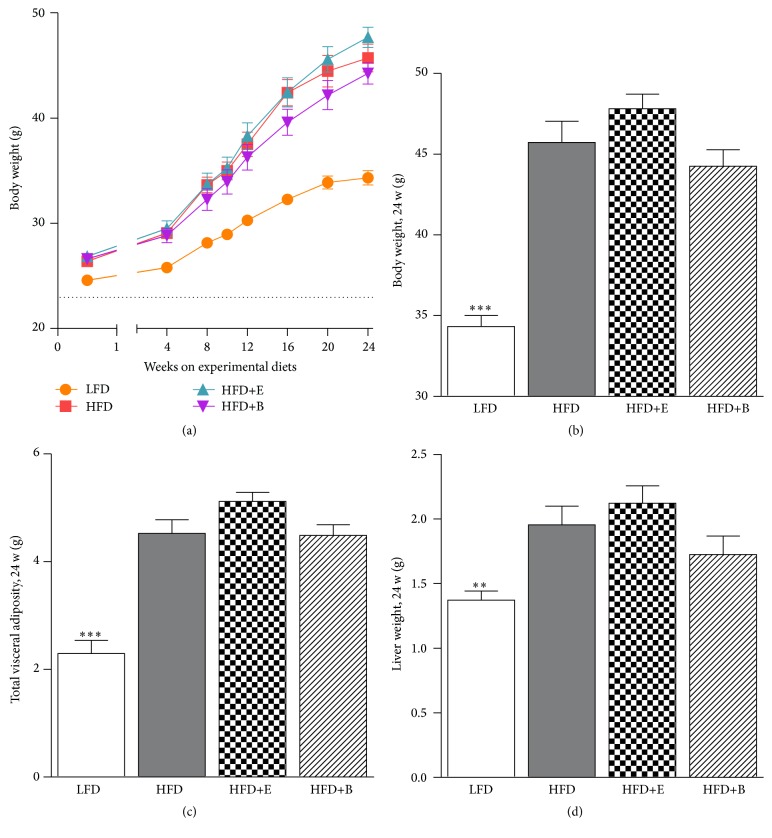
Body and organ weights. Polyphenol supplementation does not attenuate HFD-induced obesity (baseline 22.95 ± 0.18) (a-b), total weight of visceral adipose depot (c), or total liver weight. Dotted line depicts baseline value. Significant (*p* < 0.05) from HFD (^*∗∗*^ *p* < 0.01, ^*∗∗∗*^ *p* < 0.001).

Time-course analysis of body weight showed a gradual increase in body weight in all experimental groups. HFD mice had a significantly higher body weight already at 3 days after the dietary switch and all subsequent time points thereafter (Figure 1(a)). The body weights at sacrifice of the HFD+E and HFD+B mice did not significantly differ from HFD mice (Figure 1(b)). At sacrifice weights of the visceral adipose depot (total of gonadal, mesenteric, and perirenal depot weights) and liver were significantly elevated in HFD mice, but no significant differences were observed in the HFD+E and HFD+B groups compared to HFD alone (Figures 1(c) and 1(d)).

### 3.2. Polyphenols Do Not Affect HFD-Induced Changes in Metabolite Utilization

**Figure 2:**
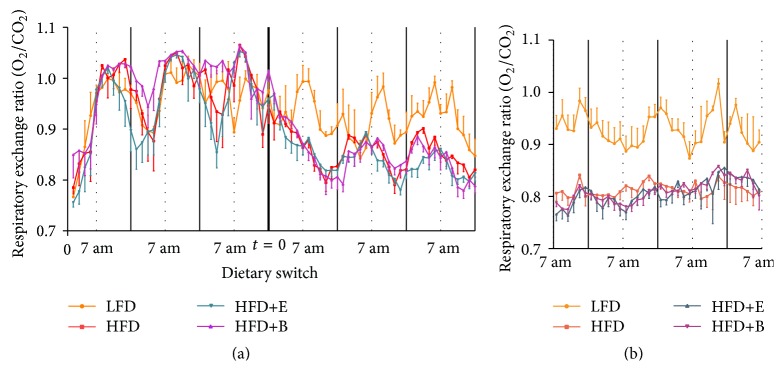
Metabolite utilization. Respiratory exchange ratio (RER; O_2_ consumed/CO_2_ produced) measured before and after dietary switch (a) and after 23 weeks (b) shows distinctive drop for all HFD groups in both the short and long term which is not enhanced by polyphenol supplementation.

The respiratory exchange ratio (RER) was calculated from indirect calorimetric data obtained at the time of dietary switch and at the end of the experiment. Switching to a HFD resulted in an immediate decline of the RER (Figure 2(a)) and remained lowered at the end of the experiment (Figure 2(b)), suggesting that fat was utilized as the main energy substrate. Short-term as well as long-term polyphenol supplementation did not affect RER compared to HFD. Notably, switching diets did also not alter intake of food pellet or physical activity in any of the experimental groups (data not shown).

### 3.3. Polyphenols Do Not Ameliorate Obesity-Induced Changes in Lipid Metabolism

**Figure 3:**
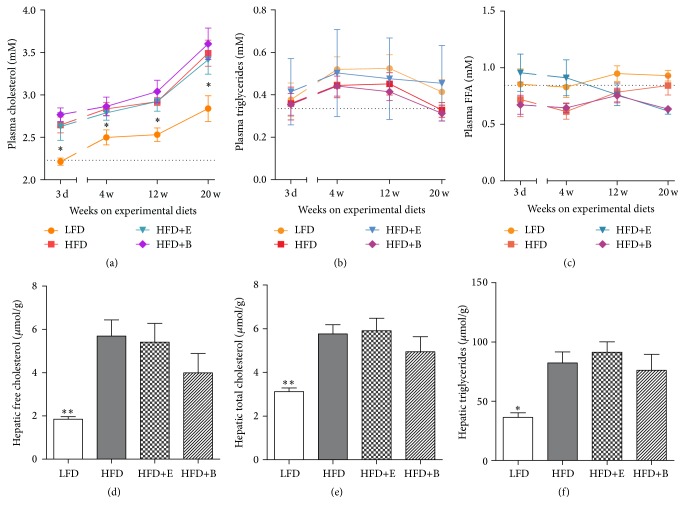
Systemic and hepatic lipids. Serial plasma (a) cholesterol (2.23 ± 0.04 at baseline), (b) triglycerides (0.34 ± 0.04 at baseline), and (c) free fatty acids (0.84 ± 0.06 at baseline), as well as hepatic (d) free cholesterol, (e) total cholesterol, and (f) triglyceride content, are not affected by polyphenol supplementation. Dotted lines depict baseline values. Significant from HFD (^*∗*^ *p* < 0.05, ^*∗∗*^ *p* < 0.01).

Plasma cholesterol levels increased in serially collected samples of all groups. HFD-feeding led to elevated cholesterol levels already after 3 days after the dietary switch and remained significantly elevated throughout the duration of the experiment. Cholesterol levels in the HFD+E and HFD+B groups did not significantly differ from the HFD group (Figure 3(a)). Similarly, plasma triglyceride and FFA levels did not show differences between the experimental groups and the HFD group (Figures 3(b) and 3(c)). Additional analysis on intrahepatic lipids levels, as assessed in liver homogenates after 24 w, showed enhanced levels of total and free cholesterol (Figures 3(d) and 3(e)) and hepatic triglycerides (Figure 3(f)) in response to HFD. Comparable intrahepatic lipid levels were observed between HFD and the groups supplemented with polyphenols. Notably, further characterization of hepatic TGs did not show a significant effect of polyphenol supplementation on any of the TG species that were assessed (Supplementary Table 1).

### 3.4. Polyphenols Do Not Prevent Obesity-Induced Systemic Inflammation

**Figure 4:**
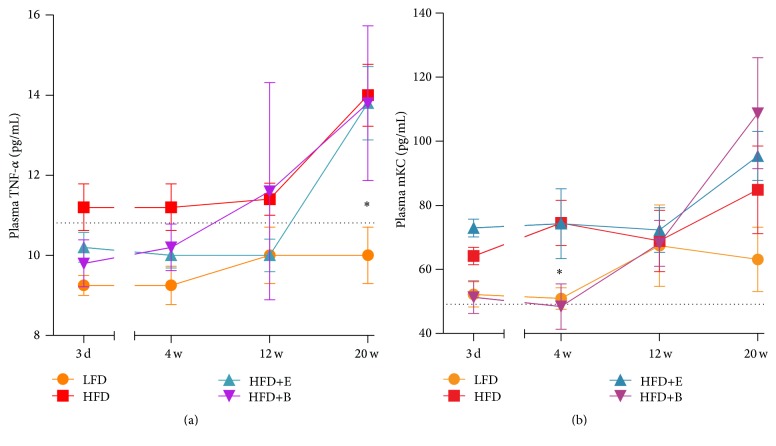
Systemic proinflammatory mediators. (a) Polyphenols do not prevent the HFD-induced increase in levels of TNF-*α* as measured in serial plasma samples (10.50 ± 0.29 at baseline). (b) Plasma levels of the neutrophil chemoattractant mKC are suppressed in the HFD+B group until 4 w but increase thereafter (49.00 ± 3.63 at baseline). Dotted lines depict baseline values. Significant from HFD (^*∗*^ *p* < 0.05).

Serial plasma cytokine analyses demonstrated elevated levels of the proinflammatory cytokine TNF-*α* shortly after switching to a HFD. This difference reached statistical significance after 20 weeks of HFD-feeding compared to LFD. No differences were observed when comparing HFD+E and HFD+B with HFD (Figure 4(a)). For mKC, a neutrophil chemoattractant in mice, an early increase was observed in HFD mice as well, which was significantly different at 4 weeks (Figure 4(b)). No differences in the HFD+E group were observed. Interestingly, mKC levels in HFD+B treated mice were similar to LFD after the dietary switch and significantly lower compared to HFD at 4 weeks. After 12 weeks mKC levels increased and levels became comparable with those of HFD controls, indicating that anthocyanin treatment retarded mKC induction. For IL-6, IFN-*γ*, IL-10, and SAA no significant differences between any of the groups were observed (Supplementary Figures 3A–3D). Plasma levels of IL-5, IL-1*β*, IL-12p70, and IL-4 were below the detection limit of the assay.

### 3.5. Polyphenols Do Not Attenuate Histopathological Features or Inflammatory Gene Expression in AT

**Figure 5:**
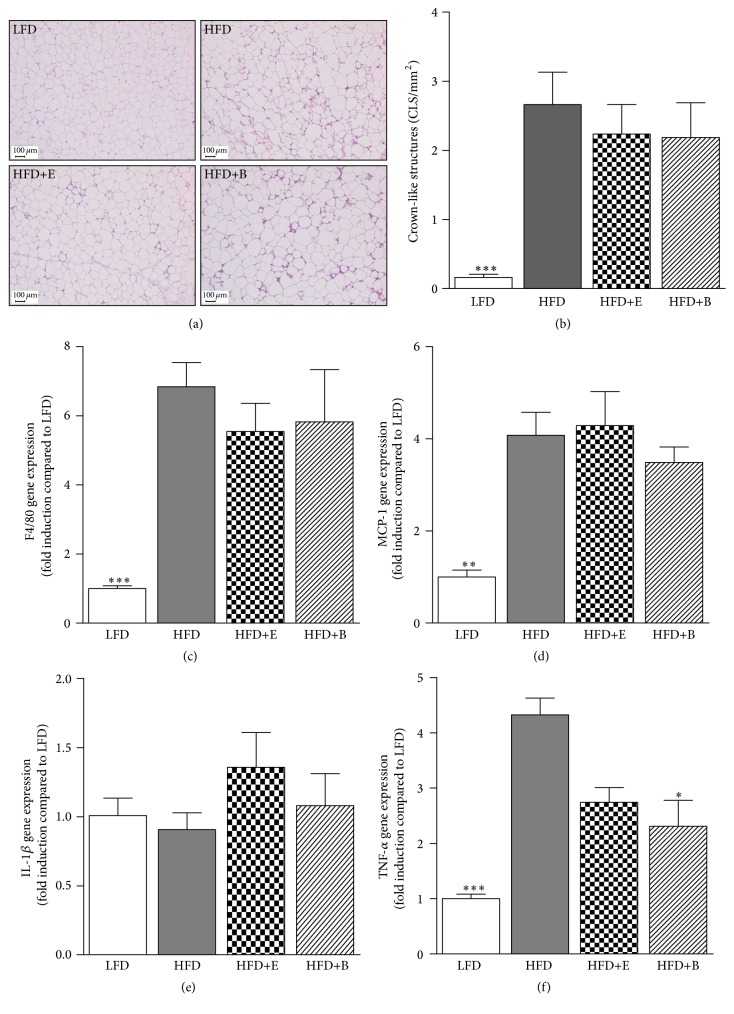
Adipose tissue inflammation at sacrifice. (a) Representative histological pictures of the visceral (gonadal) adipose tissue from different groups demonstrating an increase in the number of crown-like structures upon HFD-feeding which is not attenuated by polyphenols supplementation. (b) Quantification of crown-like structures (c) F4/80 and (d) MCP-1 gene expression confirm this phenotype. Expression of the IL-1*β* gene is not affected (e) in contrast to* Tnf* (f) which is significantly lower in the HFD+B group compared to HFD. Significant from HFD (^*∗*^ *p* < 0.05, ^*∗∗*^ *p* < 0.01, and ^*∗∗∗*^ *p* < 0.001).

Histological assessment of the visceral (gonadal) AT depot (Figure 5(a)) showed increased numbers of crown-like structures (CLS; Figure 5(b)) in all HFD groups. Neither HFD+E nor HFD+B differed significantly from HFD in number of CLS. The CLS data were in line with gene expression analyses of the macrophage marker F4/80 (Figure 5(c)) and monocyte chemoattractant factor MCP-1 (Figure 5(d)). mRNA expression of IL-1*β* was not affected by any of the experimental diets (Figure 5(e)). Interestingly, mRNA expression of TNF-*α* was 4-fold higher in HFD mice compared with LFD but only 2-3-fold higher in HFD+E and HFD+B mice when compared to LFD mice (Figure 5(f)). Moreover, TNF-*α* expression was significantly lower in HFD+B mice compared to HFD.

## 4. Discussion

Here we investigated the potential beneficial effects of anthocyanin and flavanol compounds on lipid metabolism (intrahepatic and systemic) and local (adipose tissue) and systemic inflammation in the context of diet-induced obesity. Though these compounds are assumed to have beneficial effects on the course of obesity-associated disorders,* in vivo* evidence on their effectiveness is inconsistent [27, 28]. Our current study does not show effects on circulating lipids or metabolic parameters. However, treatment with a polyphenol-rich bilberry extract resulted in a significant reduction of mKC early in time. This effect was transient and lost at the later time points. Interestingly, HFD+B persistently lowered the gene expression of TNF-*α* in the AT indicating an anti-inflammatory effect that persisted.

Various studies have shown the antiobesity effect of compounds from the anthocyanin and catechin subclasses of polyphenols* in vivo *[29, 30]. This adiposity-modulating effect could have multiple origins. First, polyphenol substances could positively affect the uptake of lipid species from the intestine by manipulation of the microbiota composition [31]. Furthermore polyphenols have been shown to enhance *β*-oxidation in the liver, increasing the utilization of fatty acids as an energy source [30]. In the AT polyphenols have been shown to inhibit adipocyte proliferation and TG synthesis while stimulating lipolysis, resulting in a reduction in adipocyte size and number [32]. Collectively, these mechanisms could contribute to a lowering of local and systemic lipid levels thereby preventing ectopic lipotoxicity. In our study we did not observe effects of polyphenols on adiposity, substrate utilization (respiratory exchange ratio), or hepatic lipid quantity and composition. The absence of an effect on plasma lipids is consistent with a recent study in which a bilberry extract and specific polyphenols (e.g., epicatechin) were analyzed for potential lipid-modulating effects in a well-established model of human dysbetalipoproteinemia, ApoE^*∗*^3-Leiden mice [21, 22].

Besides lipid-modulating effects (which may indirectly prevent metabolic inflammation by alleviating metabolic overload), polyphenols have also been postulated to have direct anti-inflammatory effects. Both anthocyanin and catechin species have been shown to prevent inflammation by inhibiting NF*κ*B activity [21, 33], a master regulator of inflammatory gene expression. Here we show that HFD-induced obesity gradually enhanced systemic TNF-*α* levels over a period of 20 weeks and found that supplementation of the HFD with the polyphenol compounds did not affect systemic TNF-*α* levels. However,* Tnf*  mRNA expression in the AT was markedly reduced (by approximately 50%) in the HFD+E and HFD+B groups compared to that in HFD (although statistical significance was only reached in the HFD+B group), indicating that the compounds have local anti-inflammatory effects at tissue level in the AT. It is thus possible that the tissue concentrations of the employed polyphenols (and their metabolites) are sufficiently high to quench inflammation but that the plasma TNF-*α* concentrations are not determined by AT, at least under the conditions employed. In line with this notion, we observed no effect on the plasma concentrations of proinflammatory cytokines upon surgical removal of inflamed AT in HFD-treated C57BL/6 mice under comparable conditions as herein [5] and human studies that investigated the release of cytokines by AT excluded visceral fat as a significant source of proinflammatory mediators including TNF-*α* [34]. The absence of an effect on TNF-*α* plasma levels suggests that other sources of TNF-*α* (e.g., immune cells) are not quenched by the polyphenol treatments. The liver as a potential source of inflammation can be excluded under the conditions employed. As we have shown previously, overt hepatic inflammation does not occur until 40 weeks of HFD-feeding in this specific mouse model of DIO [23] and is therefore unlikely to have contributed to circulating TNF-*α* levels.

While infiltrating macrophages are considered the main contributors to chronic AT dysfunction, neutrophils may also play an important role, particularly during the initial onset of the inflammatory response [35]. Therefore, inhibiting neutrophil infiltration could be beneficial in the initial course of obesity. Recent studies have shown that bilberry extract markedly reduced hepatic neutrophil infiltration in nonalcoholic steatohepatitis (NASH) [22] and catechins have been shown to reduce the inflammatory potential of neutrophils in obesity [36]. We observed that HFD-feeding was associated with a significant elevation in plasma levels of the neutrophil chemoattractant mKC within the first 4 weeks after the dietary switch. Interestingly, we found that bilberry extract, but not epicatechin, significantly attenuated this early increase. After 12 weeks however, mKC levels in the HFD+B group were similar to those in HFD and HFD+E fed mice, suggesting that short-term metabolic inflammatory responses can be quenched by bilberry extract but that these dietary components are not powerful enough to reduce a long-term metabolic stress from HFD. Though not assessed in this study, the early attenuating effect on mKC may have led to lower numbers of neutrophils. This may potentially explain the reduced AT inflammation (i.e., reduced* Tnf* mRNA expression) after 24 weeks as it may have delayed the recruitment of macrophages.

A potential explanation for the discrepancy between the positive health effects of polyphenols reported by others and the present study may be the higher dosages applied in such studies [37, 38]. As higher intake results in higher plasma levels of polyphenol metabolites, effects are likely to be greater as well although such pharmacological dosages may also be constrained by the occurrence of negative side effects [39, 40]. Herein, we notably aimed to investigate the effects of polyphenols at a moderate dose that can be translated to the situation in humans. Differences in experimental diets used may also explain observed differences in efficacy. For instance, it should be noted that an unhealthy obesogenic human diet not only contains high levels of dietary fat, as was studied here, but often contains high levels of cholesterol as well. As already small quantities of dietary cholesterol mixed with HFD can have pronounced inflammatory effects compared with HFD alone [41] and since dietary cholesterol may affect organs different than those that dietary fat does, it can be postulated that polyphenols may have different effects on HFD-induced inflammation when compared to high-cholesterol diet- (HCD-) induced inflammation. Therefore, future studies that simultaneously examine HFD- and HCD-induced inflammation and the effects of polyphenol supplementation thereupon could shed light on this topic.

There is a rapidly growing body of literature on putative beneficial effects of polyphenols in obesity and associated disorders. These studies hardly investigate long-term effects of polyphenols, and they often use relatively high (supraphysiological) dietary polyphenol concentrations in combination with experimental diets that are difficult to translate to humans. In the present long-term intervention study with (−)-epicatechin or an anthocyanin-rich bilberry extract (employed at translational doses) we found no effects on plasma lipids and effects on obesity-associated inflammation. Anti-inflammatory effects were either transient (e.g., mKC) or tissue-specific (e.g., TNF-*α* in AT) and these tissue-specific effects were not reflected in plasma. In conclusion, long-term dietary supplementation with polyphenols attenuates specific aspects of diet-induced metabolic stress. The assessment of these effects however requires an analysis on tissue level.

## Supplementary Material

Supplementary material shows schematic representation of the study's design and the analyses that were performed (Suppl. Fig. 1). Furthermore the average caloric intake per day as measured over seven days is shown for three time points and depicts that similar caloric intake was observed in HFD, HFD+E and HFD+B groups (Suppl. Fig. 2). Other inflammatory markers that were assessed but did not show any significant differences between the diets are shown. Serial plasma levels of IL-6, IFN-y, IL-10 and SAA show not to be affected by HFD-feeding or by polyphenol supplementation (Suppl. Fig. 3). Lastly, hepatic triglyceride profile at sacrifice is shown. In-depth analysis of hepatic liver lysates shows no significant effect of polyphenol supplementation on any of the fatty acid groups assessed (Suppl. Table 1).
