# Supplementary material for: Effects of Anthocyanin and Flavanol Compounds on Lipid Metabolism and Adipose Tissue Associated Systemic Inflammation in Diet-Induced Obesity
Source: Mediators Inflamm. 2016 Jun 6;2016:2042107. doi: 10.1155/2016/2042107 (PMC4913062; doi:10.1155/2016/2042107)
Supplement: Supplementary file 1 — Supplementary material shows schematic representation of the study's design and the analyses that were performed (Suppl. Fig. 1). Furthermore the average caloric intake per day as measured over seven days is shown for three time points and depicts that similar caloric intake was observed in HFD, HFD+E and HFD+B groups (Suppl. Fig. 2). Other inflammatory markers that were assessed but did not show any significant differences between the diets are shown. Serial plasma levels of IL-6, IFN-y, IL-10 and SAA show not to be affected by HFD-feeding or by polyphenol supplementation (Suppl. Fig. 3). Lastly, hepatic triglyceride profile at sacrifice is shown. In-depth analysis of hepatic liver lysates shows no significant effect of polyphenol supplementation on any of the fatty acid groups assessed (Suppl. Table 1). [file 2042107.f1.docx]

**
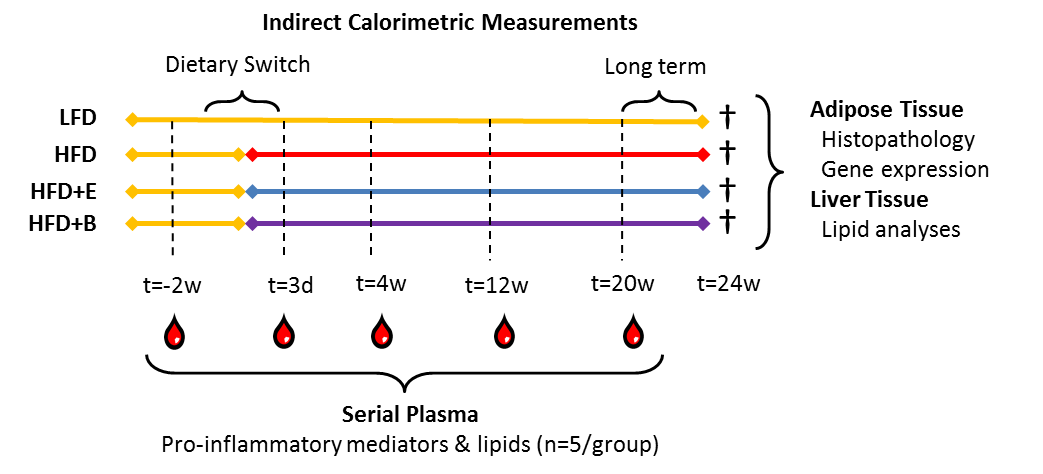
**

**Supplementary Figure 1. Experimental Design.** Schematic representation of study design and analyses that were performed

**
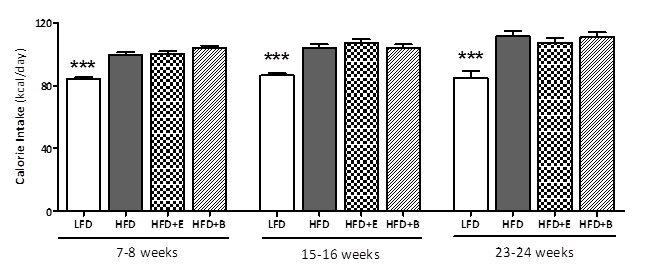
**

**Supplementary Figure 2. Caloric intake over time.** Average caloric intake per day as measured over seven days at three time points shows similar caloric intake in HFD, HFD+E and HFD+B groups.

**
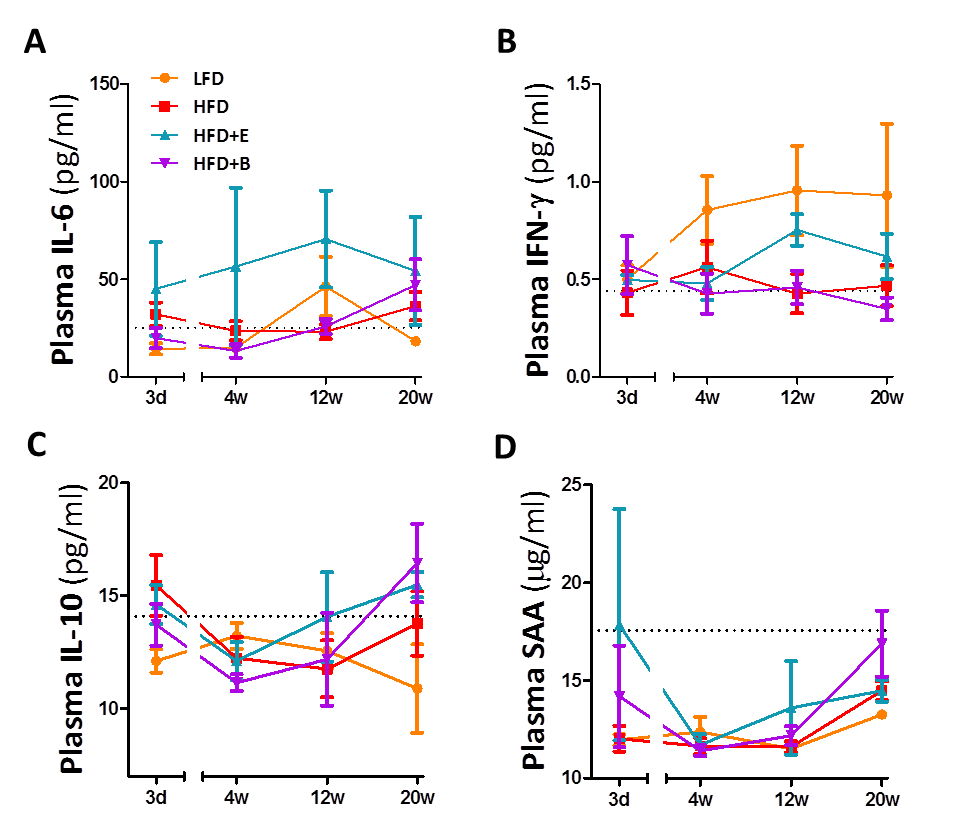
**

**Supplementary Figure 3. Other systemic inflammatory markers.** Serial plasma levels of (A) IL-6 (25.00±6.68 at baseline), (B) IFN-y (0.45±0.05 at baseline), (C) IL-10 (14.25±0.48 at baseline) and (D) SAA (17.17±2.16) are not affected by HFD-feeding or by polyphenol supplementation. Dotted lines depict baseline values.

**
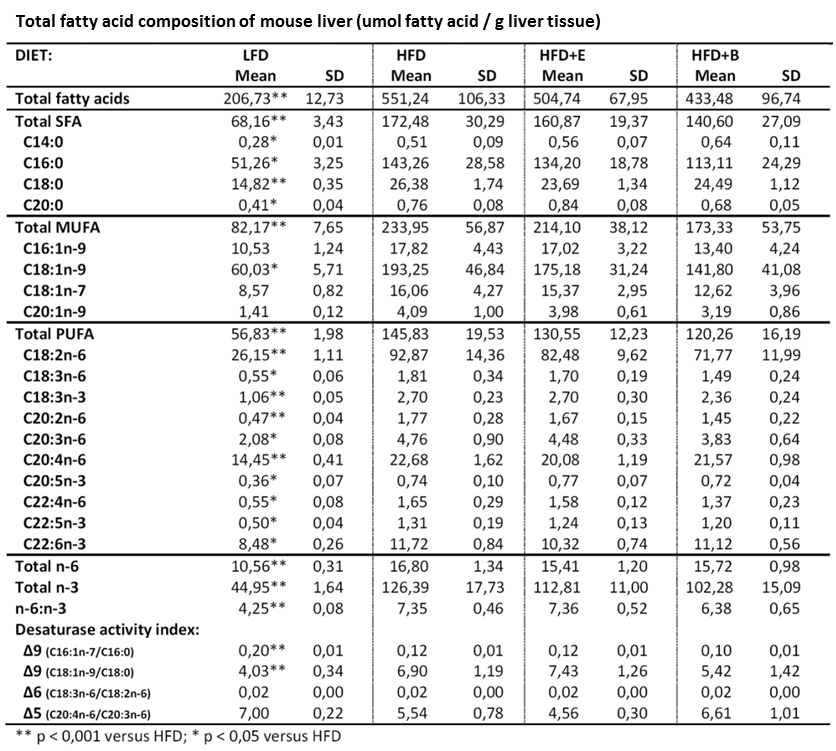
**

**Supplementary Table 1. Hepatic triglyceride profile at sacrifice.** In-depth analysis of hepatic liver lysates shows no significant effect of polyphenol supplementation on any of the fatty acid groups assessed.
